# Late-onset seizures and epilepsy: Electroclinical features suggestive of autoimmune etiology

**DOI:** 10.3389/fneur.2022.924859

**Published:** 2022-08-12

**Authors:** Alessandra Morano, Emanuele Cerulli Irelli, Enrico Michele Salamone, Biagio Orlando, Martina Fanella, Emanuele Tinelli, Gabriele Ruffolo, Luigi Zuliani, Jinane Fattouch, Mario Manfredi, Anna Teresa Giallonardo, Carlo Di Bonaventura

**Affiliations:** ^1^Department of Human Neurosciences, Epilepsy Centre, “Sapienza” University of Rome, Rome, Italy; ^2^Neurology Unit, Ospedale “Fabrizio Spaziani”, Frosinone, Italy; ^3^Unit of Neuroradiology, Department of Medical and Surgical Sciences, “Magna Graecia” University, Catanzaro, Italy; ^4^Department of Physiology and Pharmacology, Istituto Pasteur-Fondazione Cenci Bolognetti, “Sapienza” University of Rome, Rome, Italy; ^5^Istituto di ricerca e cura a carattere scientifico (IRCCS) San Raffaele Roma, Rome, Italy; ^6^Neurology Unit, AULSS8 Berica, Vicenza, Italy

**Keywords:** late-onset epilepsy, autoimmune encephalitis, late-onset seizures, elderly, ambulatory EEG, piloerection, temporal lobe, sleep

## Abstract

**Introduction:**

Late-onset epilepsy (LOE) has recently become a topic of intense research. Besides stroke, tumors, and dementia, autoimmune encephalitis (AE) has emerged as another possible cause of recurrent seizures in the elderly, and may account for a proportion of cases of LOE of unknown origin (LOEUO). This 24-h ambulatory electroencephalography (AEEG)-based study compared patients with LOEUO and AE to identify features suggestive of immune-mediated seizures in the elderly.

**Materials and methods:**

We retrospectively reviewed 232 AEEG examinations performed in patients over 55 years with ≥6-month follow-up, and selected 21 subjects with AE and 25 subjects with LOEUO. Clinical charts and AEEG recordings were carefully analyzed.

**Results:**

Twenty-five patients with LOEUO (12 women, mean age at onset 67.9 years) and 21 AE subjects (8 women, mean age at onset 65.7 years) were enrolled. High-frequency seizures were reported in 20/21 AE and 7/25 LOEUO cases (*p* < 0.00001). Focal aware seizures were more common in AE (14/21 vs. 6/25, *p* = 0.00058), whereas “isolated” focal-to-bilateral tonic-clonic seizures occurred in 5/25 patients with LOEUO only (*p* = 0.053). AE subjects reported ictal autonomic manifestations more frequently (*p* = 0.0033). Three-hundred-seventy and 24 seizures were recorded in 13/21 patients with AE and 3/25 patients with LOEUO, respectively (*p* = 0.0006). Interictal epileptiform discharges were observed in 70% of both groups, but their sleep activation was more common in AE (*p* = 0.06).

**Conclusion:**

Our study shows that high-frequency focal seizures with autonomic manifestations should raise the suspicion of AE in the elderly with new-onset seizures. It also highlights the relevant contribution of AEEG, which might reduce the diagnostic delay and provide useful clues to recognize AE.

## Introduction

Late-onset epilepsy (LOE) has recently become a topic of great interest for both the clinicians and researchers. This surge of attention first stems from the mere incidence of epilepsy among older individuals (with reported annual rates of 135 per 10^5^ people over 80 years), which is destined to rise further due to increased life expectancy (1, 2). In addition, the elderly represents a “special” population given their comorbidities, frailty, and peculiarities such as the higher risk of drug–drug interactions and heightened sensitivity to adverse events (2). Finally, LOE puts the spotlight on the reciprocal relationships between epilepsy and other neurological disorders, particularly stroke and dementia, that are far from being fully elucidated (3, 4). Cerebrovascular diseases (CVDs) currently represent the leading cause of LOE (accounting for up to 50% of the cases with a known etiology), followed by neurodegenerative conditions (10–20%), traumatic brain injuries (up to 25%), and brain tumors (10–30%). Still, a precise cause cannot be identified in a remarkable proportion of cases (25–53% according to different series) (5, 6), currently defined as LOE of unknown origin (LOEUO) or nonlesional LOE. In recent years, autoimmune encephalitis (AE) has also emerged as a potential cause of seizures in adult and elderly individuals, and this might have a dramatic impact on LOE epidemiology and diagnostic approach. Indeed, despite being considered a rare entity, AE is a polymorphic and widely underrecognized disease that may account for a relevant proportion of older patients with otherwise unexplained recurrent seizures/epilepsy. In this context, the timely suspicion of an underlying immune-mediated condition is crucial to start immunotherapy (IT) and minimize long-term sequelae, including cognitive impairment and autoimmune-associated epilepsy. Unfortunately, AE diagnosis can be challenging, especially in milder cases or when supporting paraclinical findings are lacking, even more so in the elderly, whose seizures are often misreported or misinterpreted. This 24-h ambulatory electroencephalography (AEEG)-based retrospective study compared the electroclinical characteristics of patients with LOEUO over 55 years with patients with AE of the same age, in order to try and outline distinct phenotypes and identify features possibly suggesting an immune-mediated origin of seizures in the elderly.

## Materials and methods

### Patients' selection

By screening all the AEEG recordings performed in the neurophysiology laboratory of Policlinico “Umberto I” of Rome from January 2008 to August 2021, we retrospectively identified 232 individuals aged ≥ 55 years at the time they undertook the examination for new-onset seizures or paroxysmal phenomena of suspected epileptic nature. The cutoff age of 55 years was chosen based on recent literature on LOE (7, 8). As a second step, through the careful review of clinical charts and neuroimaging studies (brain MRI or—if not available—CT scans), we selected two populations of patients diagnosed with LOEUO and AE, respectively. The inclusion criteria for the LOEUO group were as follows: (1) ≥ 1 AEEG examination performed early during the diagnostic workup for new-onset seizures; (2) absence of MRI-/CT-detected brain lesions, such as ischemic strokes, intracranial hemorrhages, tumors, posttraumatic lesions, and focal atrophy, causally related to seizures based on anatomoclinical correlations (conversely, individuals with structural abnormalities such as arachnoid cysts and meningiomas, judged as “incidental findings” by the investigators, were included); (3) no former diagnosis of dementia; and (4) Fazekas score < 2 (9). In AE cases, the diagnosis was confirmed according to the recent criteria by Graus et al. (10), and only individuals with: (1) ≥ 1 AEEG examination performed early during the diagnostic workup for new-onset seizures, and (2) a history of seizures other than faciobrachial dystonic seizures (FBDS) was enrolled. The study exclusion criteria were: (1) incomplete clinical documentation, and (2) follow-up duration < 6 months.

The study was approved by the local Ethics Committee.

### General data collection

The patient's demographics and clinical data were collected, specifically: sex, age at seizure onset, diagnostic delay, cerebrovascular (CV) risk factors, previous and/or concomitant malignancies, and follow-up duration. As to the AE group, laboratory findings were also reviewed, with particular attention to the results of the autoantibodies (Abs) screening performed on either cerebrospinal fluid (CSF) or serum during the diagnostic workup. According to our institution's common practice, all the patients with AE undertook the search for Abs directed against intracellular (i.e., anti-Hu, Yo, Ri, amphiphysin, CRMP5/CV2, Ma2) and neuronal surface antigens [i.e., N-methyl-D-aspartate receptor (NMDAR), leucine-rich glioma-inactivated protein 1 (LGI1), contactin-associated protein-like 2 (CASPR2), alpha-amino-3-hydroxy-5-methyl-4-isoxazole-propionate receptor (AMPAR), γ-aminobutyric acid receptor (GABAR)_B_]; conversely, anti-glutamic acid decarboxylase (GAD) 65 and anti-GABAR_A_ Abs were tested only in a minority of subjects.

The type and semiology of the reported seizures were carefully reviewed, along with their frequency (defined as “high” when seizures recurred more than once per month), episodes of status epilepticus (SE), number of trials with antiseizure medications (ASMs), and seizure outcome. In the case of SE at presentation, the seizure frequency was considered “high” per definition. Seizures were classified as focal aware seizure (FAS), focal impaired awareness seizure (FIAS), and focal to bilateral tonic-clonic seizure (FTBTCS) according to the latest International League Against Epilepsy (ILAE) proposal (11). As to the ictal semiology, the occurrence of autonomic manifestations, and piloerection, in particular, was specifically analyzed. In addition, based on the investigators' global interpretation of ictal and peri-ictal signs and symptoms, seizures were classified as probably originating from the temporal lobe or not (i.e., extratemporal or not localizable). For the study purpose, seizure freedom was defined as the absence of seizures for at least 6 months at the last follow-up visit.

### Analysis of ambulatory electroencephalography findings

At least one 24-h AEEG (Micromed System Plus, Treviso, Italy) with reduced electrode montage (9 electrodes, high-pass filter: 1.6 Hz, low-pass filter: 70 Hz, and sensitivity: 10 μV/mm) was available for all the study participants. In those who performed more than one AEEG examination, only the earliest was considered. The recording duration ranged from 21 to 25 h in the whole study population, with a median length of 22.4 h. The following interictal EEG features were collected and analyzed: (1) focal/diffuse slowing; (2) interictal epileptiform discharges (IEDs), in terms of presence, localization, and lateralization; and (3) IED frequency during wakefulness and sleep. Indeed, to provide a more precise evaluation of IEDs across different brain states, for all the tracings but one (i.e., a patient with almost subcontinuous nocturnal seizures was excluded from analysis), two 1-h epochs were selected in wakefulness and sleep, respectively, and the exact number of IEDs in each epoch was counted by two independent reviewers (possible discrepancies were resolved through discussion with an additional reviewer). The sleep epoch was selected within the first sleep cycle (starting from the first identified spindle), based on previous studies (12). The wakefulness epochs had the minimum possible level of background artifacts. The “sleep activation” of interictal abnormalities was defined as an increase by 100% in IED number during sleep compared with wakefulness; if no IED at all was identified during the chosen wakefulness epoch, the detection of ≥10 IEDs during the sleep one qualified as “sleep activation.” Finally, interictal abnormalities were classified as bilateral when the proportion of asynchronous IEDs exceeded 10% (13).

Seizures recorded during AEEG were counted and analyzed, with particular focus on their localization, their circadian distribution, and their clinical correlates, as reported in the patients' log. A subclinical seizure (SCS) was defined as the occurrence of a paroxysmal electrographic pattern consistent with a seizure (i.e., showing a plausible electrographic field and a proper temporal-spatial evolution in frequency, amplitude, and morphology) that was not associated with identifiable clinical manifestations other than very subtle signs (i.e., microarousals during sleep, unreported changes in the heart rate).

### Statistical analysis

Data were tested for normal distribution using the Shapiro–Wilk test, and presented as mean (SD) and median [interquartile range (IQR)] as appropriate. The comparison between the AE and LOEUO groups was performed through the Mann–Whitney *U* test in case of continuous variables (e.g., age at onset, diagnostic delay, number of CV risk factors, ASM trials, follow-up duration), whereas categorical data (e.g., sex, history of neoplasms, seizure type and origin, presence of ictal autonomic manifestations and piloerection, AEEG-detected seizures, IED sleep activation, bilateral IEDs) were compared through the Fisher's exact test or chi-squared test. Group tests were two-sided with *P* < 0.05 that considered statistically significant. To evaluate the independent correlation between the investigated electroclinical variables and seizure autoimmune etiology, a multivariate logistic regression model was elaborated. Variables showing a *p*-value < 0.05 at univariate analysis (the Fisher's exact test or chi-squared test for normal variables and the Mann–Whitney *U* test for continuous variables, as previously illustrated) were included in the multivariate model. Due to the rarity of the analyzed condition (i.e., AE), which resulted in a limited number of events, a penalized logistic regression with Firth's correction was applied to reduce the bias related to the small sample size. The diagnosis of AE served as the dependent variable. IBM SPSS Statistics version 25 for Windows (IBM Incorporation, Armonk, New York, USA) was used for data analysis.

## Results

### Patients' demographics and general data

Overall 46 subjects were considered eligible for the study: 25 patients identified as LOEUO (12 women, mean age at onset 67.9 ± 7.25 years) and 21 individuals diagnosed with AE (8 women, mean age at onset 65.7 ± 7.39 years). The latter group included 6 patients with specific Abs (i.e., anti-LGI1 and anti-CASPR2 Abs in 2 subjects each, anti-GABA_B_R and anti-Hu/Ri in 1 subject each); in the remaining 15 seronegative cases, the diagnosis was confirmed based on the criteria by Graus et al. (10), according to the methods: specifically, a diagnosis of “definite” AE was achieved in overall 12/21 patients, whereas 9 patients were classified as “possible” AE. Although inflammatory CSF changes were detected in a minority of patients with AE (5/21), MRI alterations suggestive of encephalitis were documented in all the cases but one (Supplementary Table 1). The two study groups, i.e., AE and LOEUO, were comparable in terms of sex distribution, age at seizure onset, history of malignancies, and presence and number of CV risk factors (Table 1). The diagnostic delay did not significantly differ between patients with AE and LOEUO (median duration: 4.5 vs. 9 months, *p* = 0.1902), although it was shorter in the former. Conversely, the follow-up period in AE was slightly longer than in LOEUO (median FU duration: 40 vs. 24 months), but such difference did not reach either statistical significance (*p* = 0.067). When retrospectively applying the Antibody Prevalence in Epilepsy and Encephalopathy (APE2) (16) score to the study population, we found that 16/21 AE cases achieved ≥ 4 points (range 3–10, median 4), whereas all the patients with LOEUO scored < 3; however, such difference was partly influenced by the inclusion criteria for LOEUO, which prevented us from attempting a proper comparison between the study groups. All the participants tried at least one ASM, and all but one patient with AE received IT as well. At the last follow-up visit, a significantly higher proportion of individuals with AE had tried ≥ 3 ASMs compared with LOEUO (6/21 vs. 1/25, *p* = 0.0365). Still, at that time, 17/21 patients with AE and 23/25 patients with LOEUO were seizure-free (*p* = 0.389).

**Table 1:** General characteristics and electroclinical features of LOEUO compared with AE.

**Patients'** **characteristics**	**LOEUO (14)**	**AE (15)**	***P*-value**
**Gender**			
Female	12	8	0.4996
Male	13	13	
Age at seizure onset (yrs), mean (SD)	67.9 (7.25)	65.7 (7.39)	0.32
History of neoplasms	4	4	1
CV risk factors	22	17	0.77
N° of CV risk factors, median [range]	1 [0–3]	1 [0–2]	
Diagnostic delay (mo), median [range]	9 [0.2–120]	4.5 [0.2–48]	0.19
Follow-up duration (mo), median [range]	24 [6-156]	40 [6-120]	0.067
**Seizure frequency, pts** ***n*** **(%)**			
High	7 (28)	20 (95.2)	**<0.00001**
Low	18 (72)	1 (4.8)	
SE	3	2	1
**Seizure type, pts** ***n*** **(%)**			
FAS	6 (24)	14 (66.7)	
FIAS	14 (56)	14 (66.7)	0.07
FTBTCS	15 (60)	8 (38)	
FTBTCS only	5 (20)	0 (0)	0.053
**Seizure semiology, pts** ***n*** **(%)**			
T lobe seizures	18 (72)	19 (90)	0.15
Autonomic ictal manifestations	7 (28)	15 (71)	**0.0033**
Piloerection	0 (0)	6 (28.6)	**0.0058**
**AEEG findings**			
Interictal abnormalities, pts *n* (%)			
Focal slowing	19 (76)	15 (71.4)	0.1237
IEDs	18 (72)	16 (76.2)	0.1039
Bilateral IEDs	5 (20)	9 (42.9)	0.09
IED sleep activation	7 (28)	11^§^ (55)	0.06
**Ictal findings**			
AEEG-recorded seizures, pts *n* (%)	3 (12)	13 (62.9)	**0.0006**
N° of recorded seizures	24	370	
Clinical/Subclinical	0/24	171/179	
Wake/Sleep	17/7	174/176	
T lobe origin, pts *n*	3	11	

AEEG, ambulatory EEG; CV, cerebrovascular; FAS, focal aware seizures; FIAS, focal seizures with impaired awareness; FTBTCS, focal to bilateral tonic-clonic seizures; IEDs, interictal epileptiform discharges; mo, months; n, number; pts, patients; SD, standard deviation; T, temporal; yrs, years. The bold values indicates statistical significance.

### Seizure characteristics

When comparing seizure characteristics, we found their frequency to be the most remarkable difference between the study groups: only 7/25 individuals with LOEUO had high-frequency seizures at the time of diagnosis compared with 20/21 patients with AE (*p* < 0.00001). Indeed, 8/25 LOEUO participants had fits recurring less than once a year, which was never the case for patients with AE. SE was either clinically observed or EEG documented in a few subjects per group (2/21 AE and 3/25 LOEUO), although this might reflect the study setting, since no patient requiring intensive care undertook AEEG in our laboratory.

The study groups appeared roughly comparable in terms of seizure types (FAS vs. FIAS vs. FTBTCS, *p* = 0.07); however, FAS was significantly more common among participants with AE than with LOEUO (14/21 vs. 6/25, *p* = 0.00058). In addition, “isolated” FTBTCS was reported in 5/25 patients with LOEUO (4 patients of whom had convulsive seizures during sleep), whereas all the AE subjects presenting FTBTCs also had FAS or FIAS (*p* = 0.053). The seizure semiology was interpreted as highly suggestive of temporal lobe origin in the vast majority of patients of both the groups (19/21 AE vs. 18/25 LOEUO, *p* = 0.15); still, when considering the occurrence of ictal autonomic manifestations, they appeared significantly more frequent among AE (15/21) than LOEUO subjects (7/25) (*p* = 0.0033), the most common being the rising epigastric sensation (in 7/21 AE cases), followed by piloerection (in 6/21 AE cases) and flushing (in 4/21 AE cases). As expected, ictal piloerection was only observed in patients with AE (6/21 vs. 0/25) (*p* = 0.0058). Conversely, in LOEUO subjects with focal seizures, cognitive manifestations, in particular dysphasia, confusion, and/or memory impairment, were the most commonly reported (in 11/25 LOEUO cases).

### Electroencephalography ictal and interictal findings

As stated in the methods, 46 AEEG examinations (one per patient) performed during the early diagnostic workup for late-onset seizures were carefully reviewed and analyzed. The focal slowing was documented in the same proportion of AE (15/21, 71.4%) and LOEUO individuals (19/25, 76%), as were IEDs [16/21 (76%) in AE vs. 18/25 (72%) in LOEUO], which were located over the temporal regions in all the cases (the concomitant involvement of either the frontal or the parietal derivations was observed in few of them). As to the lateralization, bilateral IEDs were more common in AE compared with LOEUO (9/21 vs. 5/25), although this difference did not reach statistical significance (*p* = 0.09). A similar but more evident trend was observed for IED activation during sleep, which was documented in 11/20 patients with AE compared with 7/25 LOEUO subjects (*p* = 0.06).

Overall, 394 seizures were recorded during AEEG: 370/394 in 13/21 (62.9%) individuals with AE (range: 1–259 per person, median: 1) and 24/394 in 3/25 (12%) patients with LOEUO (range: 1–21 per person, median: 0) (*p* = 0.0006). One-hundred-seventy-one of 370 ictal events documented in AE cases had clinical manifestations; in addition, 2/21 patients reported overall 6 FAS without a clear EEG correlate. Of the remaining 199 SCSs (recorded in 7/21 patients), 196 occurred during sleep. All the 24 seizures detected in 3/25 patients with LOEUO were subclinical, and were recorded either in wakefulness (17/24) or in sleep (7/24). Seizures arose from the temporal lobe in all the subjects, but 2 patients with AE, presented both the clinical and subclinical paroxysmal activities, involving, respectively, the temporoposterior and the centrotemporal derivations from the very seizure onset.

**Figure 1:**
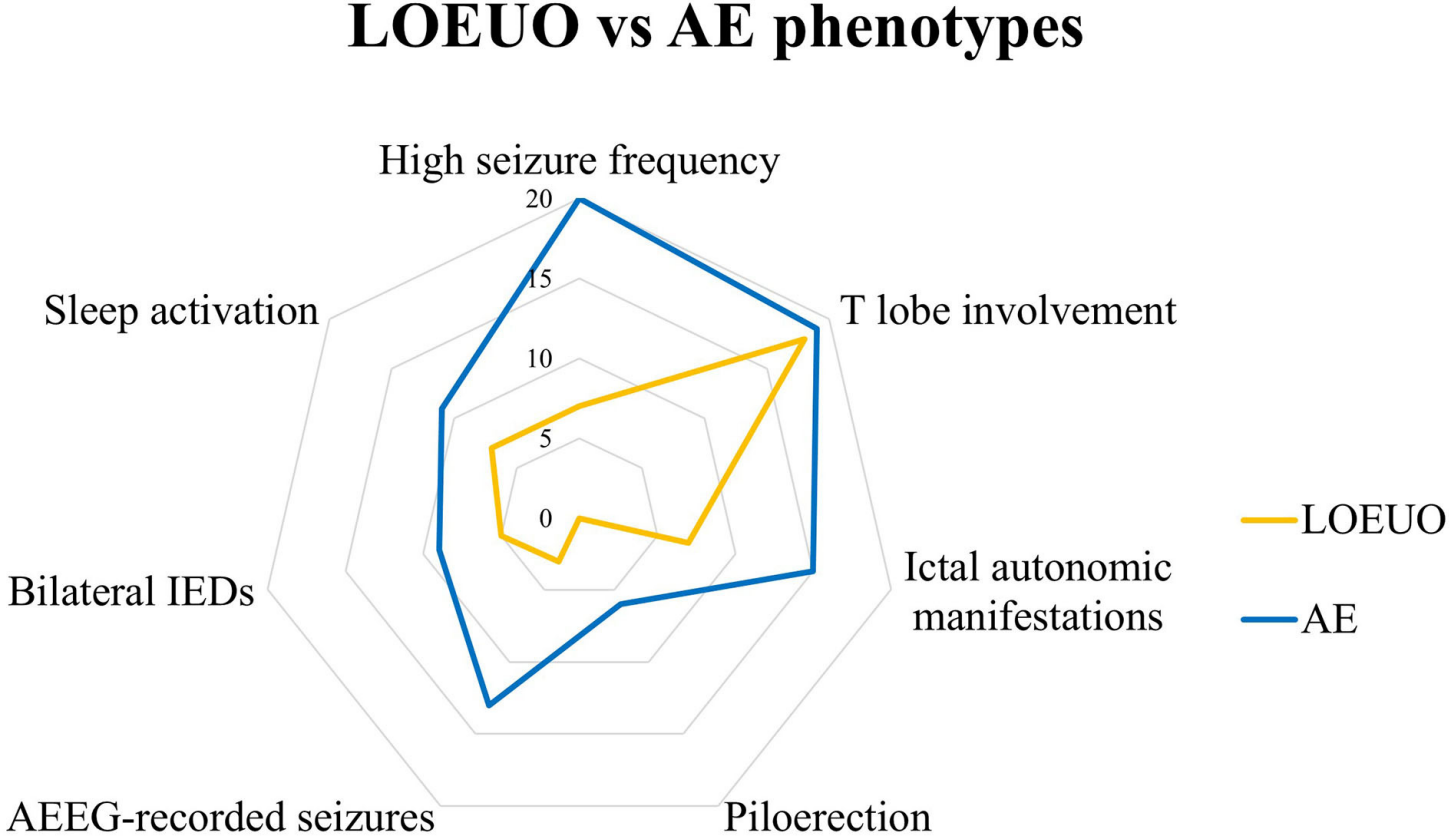
Electro-clinical profile of LOEUO vs AE. AE, autoimmune encephalitis; AEEG, ambulatory EEG; IEDs, interictal epileptiform discharges; LOEUO, Late-onset epilepsy with unknown origin; T, temporal.

The main electroclinical features analyzed for comparison between AE and LOEUO are illustrated in Table 1 and Figure 1.

At univariate logistic regression analysis, seizure frequency (high/low), ictal autonomic manifestations (present/absent), AEEG-detected SCSs (yes/no), and AEEG-detected CSs (yes/no) were found to be significantly associated with the diagnosis of immune-mediated seizures. Considering the occurrence of high multicollinearity between AEEG-detected CSs and other variables (namely high seizure frequency and SCSs), we included only AEEG-detected SCSs in the multivariate model. The analysis confirmed the independent association of the three selected variables with the diagnosis of immune-mediated seizures (Table 2).

**Table 2:** Firth's multivariate logistic regression analysis.

	**B**	**SE**	**Exp (B)**	**95% CI**	***P*-value**
AEEG-recorded subclinical seizures	1.88	1.05	6.55	1.1–70.1	0.047*
Ictal autonomic manifestations	2.09	0.94	8.1	1.47–67.76	0.015*
Seizure frequency	2.97	1.02	19.53	3.45–218.54	<0.001*

The asterisks indicate statistically significant variables (p < 0.05).

## Discussion

The increasing incidence figures of epilepsy in the elderly, along with the special needs of this patient population, have recently turned LOE into a priority on the epileptologists' agenda worldwide. In fact, epilepsy in late life is more than a practical concern for physicians: it is a crossroad of other conditions, particularly stroke and dementia, that can precede – or even follow – epilepsy, reflecting the complex reciprocal relationships between epileptogenesis, CVD, neurodegeneration, and neuroinflammation (3, 4). Such an intricate plot is destined to thicken further, due to AE recently emerging as another possible cause of seizures/epilepsy in adults and the elderly. Indeed, when no alternative structural etiology can be identified in older patients with new-onset seizures, suspecting an immune-mediated condition would have dramatic diagnostic and therapeutic implications. The recently elaborated APE2 score is a useful screening tool to identify adult patients with new-onset seizures at higher risk of harboring antineuronal antibodies; however, it could be less reliable in “milder” cases and in the elderly, whose frequent neurological (e.g., cognitive impairment, mood disorders) and extraneurological comorbidities (e.g., heart diseases, malignancies) might act as confounding factors.

Following these considerations, we retrospectively compared two cohorts of patients with AE and LOEUO with seizure onset after 55 years, and we found that they have distinctive electroclinical profiles that can be clearly outlined, especially with the help of long-term EEG.

The first and most striking difference between the study groups lay in seizure frequency, as all the patients with AE but one reported ≥ 1 seizure per month (weekly episodes in 3 cases and daily in 10 cases), compared with only 7/25 LOEUO subjects (*p* < 0.00001). Such discrepancy was matched by the different proportions of patients with AEEG-documented ictal events, remarkably higher in the former group (*p* = 0.0006), as discussed later in this section. The high seizure frequency found in AE does not come as a surprise based on previous literature (17, 18), and possibly reflects the nature of these seizures, which should be interpreted as “acute symptomatic” of the underlying immune-mediated process, according to recent recommendations (although we cannot theoretically exclude that autoimmune-associated epilepsy had already ensued in some of our patients, especially those with the longest diagnostic delay) (19). Conversely, data on LOEUO are scant, and seizure frequency in this population has hardly ever been analyzed, or even reported, so far. The low frequency documented in our study appears in line with the overall “benign” course generally described in LOE and observed in our population as well, where 17/25 subjects achieved seizure freedom on the first monotherapy. Nonetheless, such rare seizures (so rare that they recurred less than once a year in 8/25 patients with LOEUO) could hamper the diagnosis of epilepsy in the elderly, already quite challenging, and further increase the diagnostic delay, which, in fact, was longer in LOEUO than in AE (median 9 vs. 4.5 months), although not significantly.

Our study groups were roughly comparable with regard to the seizure type, although FAS was more common in AE (14/21 vs. 6/25) and FTBTCS appeared slightly more frequent in LOEUO (15/25 vs. 8/21). These findings are in line with previous articles, reporting generalized tonic-clonic seizures in 30–47% of cases of epilepsy in the elderly (6–8, 20), although proper comparisons are difficult due to differences in the study populations. In our LOEUO cohort, 5/25 patients reported FTBTCS as the only seizure type (mainly occurring at night), which was never the case among the subjects with AE (*p* = 0.053). This is an interesting trend with potential practical implications: indeed, convulsive seizures represent a most dramatic event, often leading the patient to seek medical attention for the first time, but in AE they are usually accompanied—or preceded, even for months—by high-frequency focal seizures, which instead might be lacking in LOEUO. Such observation highlights, once again, the importance of properly taking the patient's medical history, and actively looking for “minor” events, which are easily overlooked by the elderly, as well as their relatives.

When focusing our attention on seizure semiology, we found that the ictal manifestations were suggestive of temporal lobe (TL) seizures in most patients of both the cohorts, as also confirmed by the localization of IEDs and paroxysmal activities documented *via* AEEG. Although the prominent TL involvement might be well assumed in autoimmune “limbic” encephalitis, it is far from expected in nonlesional LOE. In fact, TL seizures have been described as the main seizure type in 59 patients with LOE and MRI/CT evidence of leukoaraiosis only (21). In addition, DiFrancesco et al. recently analyzed 23 subjects with LOEUO by means of EEG, PET, and neuropsychological assessment, all pointing to a TL dysfunction of variable extent (8). These findings, along with evidence showing that epileptic seizures in the elderly could be a prodrome of Alzheimer's disease (22), suggest that the temporal lobe might represent a *locus minoris resistentiae*, particularly susceptible to different kinds of injuries and/or processes underlying both the AE and LOEUO, i.e., occult cerebrovascular disease, neurodegeneration, and immune-mediated mechanisms. The reasons, unfortunately, are still to be fully disclosed: anatomical factors such as the relatively simple architecture, the proximity of the circumventricular organs, and the properties of the blood–brain barrier, as well as neuroplasticity-related mechanisms and vulnerability to neurotropic viruses, are supposed to play a role (15, 23).

Notwithstanding these basic similarities, we also found that focal seizures with autonomic manifestations were far more common in AE than in LOEUO (15/21 vs. 7/25, *p* = 0.0033). This was not accounted for by ictal piloerection alone, reported in 6/21 patients with AE and associated with other vegetative symptoms in half of them. On one hand, such observation revives pathophysiological considerations about the crucial involvement of the central autonomic network (CAN) in AE. On the other hand (more practical), it also signifies that “looking closer,” paying attention to semiological details—which should be actively sought, since they are rarely reported by the patients spontaneously—could provide physicians with important clues about the autoimmune origin of new-onset seizures in the elderly.

With respect to the EEG findings, the careful revision of the AEEG examinations undertaken by all the study participants allowed us to detect a far higher number of ictal events in a significantly larger proportion of patients with AE compared with patients with LOEUO (370 seizures in 13/21 AE cases vs. 24 seizures in 3/25 LOEUO cases, *p* = 0.0006). This observation relaunches the role of AEEG in the diagnostic workup of new-onset seizures, as well as in the follow-up of long-lasting epilepsy (24). Indeed, prolonged EEG monitoring increases the chance of recording and recognizing seizures as such—which could be particularly challenging in older people with several comorbidities and possible alternative diagnoses. Moreover, AEEG also allows to identify subclinical events, (which appeared to be significantly associated with AE diagnosis in the multivariate regresso model) which appeared to be significantly associated with AE diagnosis in the multivariate logistic regression model, and, thus, to properly evaluate the overall seizure burden, that especially in AE might easily contribute to cognitive impairment.

When analyzing the interictal findings, IEDs were found in more than 70% of patients of both the cohorts, a much higher proportion than those reported in previous articles on LOE, where it ranged from 16 to 35% (6, 8, 25). Such discrepancy likely depends on the use of long-term monitoring, including sleep recording, and could have important clinical implications: indeed, especially in patients with LOEUO with rare seizures, identifying IEDs might actually hasten the definite diagnosis of epilepsy and prompt clinicians to start ASMs, which could easily result in long-lasting seizure freedom and prevent unnecessary inconveniences and dangers (e.g., seizure-related falls). The “sleep activation” of interictal abnormalities was observed in both the study groups, as expected based on previous literature (14, 26), but appeared more common in patients with AE (11/20 vs. 7/25, *p* = 0.06), despite not reaching statistical significance, probably due to the small sample size. Although the role of sleep on IED facilitation has not been specifically addressed in AE, the increase of interictal abnormalities during nonrapid eye movement (NREM) sleep has been proved to affect memory consolidation by interfering with sleep-related thalamo-cortical-hippocampal coupling (27), and, thus, could *per se* contribute to cognitive impairment in this peculiar patient population.

This study has several limitations, the most relevant being its retrospective design, with its intrinsic risk of a selection bias, the fact that patients with LOUEO were not specifically tested for antineuronal Abs, and the limited sample size, which might have affected the significance of some analyses.

In conclusion, our study shows that, despite sharing some similarities such as seizure type and onset, older patients with AE can be distinguished from those with LOEUO based on their electroclinical features: in particular, high-frequency focal seizures with ictal autonomic manifestations in the elderly should raise the suspicion of an underlying immune-mediated disorder. Our study also highlights the relevant contribution of AEEG, a noninvasive, economic tool, which might reduce the diagnostic delay in LOEUO, and provide useful information to recognize AE.

## Data availability statement

The raw data supporting the conclusions of this article will be made available by the authors, without undue reservation.

## Ethics statement

The studies involving human participants were reviewed and approved by Sapienza University Ethics Committee. The patients/participants provided their written informed consent to participate in this study.

## Author contributions

CD: full access to all the study data, full responsibility for the integrity of the data and the accuracy of the data analysis, and study supervision. AM, EC, and CD: study concept and design. AM, EC, ES, BO, MF, GR, and JF: data collection and interpretation. ET: review and analysis of neuroimaging studies. LZ: laboratory analysis. AM: study draft. EC: statistical analysis. AM, EC, ES, BO, MF, ET, GR, LZ, JF, MM, AG, and CD: manuscript revision and critical reappraisal. All the authors have contributed to the manuscript, and read and approved the submitted version of the manuscript.

## Funding

This study has received funding from the European Union's Horizon 2020 Research and Innovation Program under Grant Agreement No. 952455. GR was supported by the Italian Ministry of Health Ricerca corrente.

## Conflict of interest

The authors declare that the research was conducted in the absence of any commercial or financial relationships that could be construed as a potential conflict of interest.

## Publisher's note

All claims expressed in this article are solely those of the authors and do not necessarily represent those of their affiliated organizations, or those of the publisher, the editors and the reviewers. Any product that may be evaluated in this article, or claim that may be made by its manufacturer, is not guaranteed or endorsed by the publisher.
