# Supplementary material for: Late-onset seizures and epilepsy: Electroclinical features suggestive of autoimmune etiology
Source: Front Neurol. 2022 Aug 12;13:924859. doi: 10.3389/fneur.2022.924859 (PMC9412019; doi:10.3389/fneur.2022.924859)
Supplement: Supplementary file 1 [file Table_1.docx]

**Supplementary Table.** General characteristics of the AE group (n 21)

| **General characteristics** |  |  |
| --- | --- | --- |
| Age at AE onset (years) | Mean, [range] | 65.7 [55-76] |
| Gender | Female (%)  Male (%) | 8 (38)  13 (62) |
| **Relevant data in medical history** | Neoplasms  Previous  Concomitant | 4  1  3 |
| **Clinical presentation at AE onset (%)**  **Diagnostic accuracy** | Seizures  Cognitive impairment  Mood/Behavioral disorders  Definite  Possible | 21 (100)  18 (85.7)  14 (66.7)  12  9 |
| **APE2 score^14^** | < 4  4 – 6  ≥ 7 | 4  12  5 |
| **Laboratory findings** |  |  |
| CSF findings | Hyperprot/Hypercell  Normal  Not available | 5  12  4 |
| AutoAbs profile (CSF/serum) | Seropositive  Anti-LGI1  Anti-CASPR2  Anti-GABA_B_R  Anti-Hu/Ri  Seronegative | 6  2  2  1  1  15 |
| **MRI findings**^¶^ | Unilateral mT  Bilateral mT  Normal | 10  10  1 |
| **Diagnostic delay** (mo), median, [range] |  | 4.5 [0.2-48] |
| **Response to Immunotherapy** | Yes/Partial  No/Not applicable | 11  10 |
| **Sequelae at follow-up** (%) | Autoimmune epilepsy  Cognitive impairment  Mood disorders | 17 (80.9)  20 (95.2)*  8 (38) |

^¶^ MRI alterations are defined as T2/FLAIR hyperintensity with or without volume changes of the mesial temporal (mT) structures

*Mild cognitive impairment in 14 patients

**Supplementary Table Legend.** AE: autoimmune encephalitis; AutoAbs: autoantibodies; CASPR2: contactin-associated protein-like 2; CSF: cerebrospinal fluid; GABA_B_R: γ-aminobutyric acid receptor; LGI1: 1eucine-rich glioma-inactivated 1; mo: months, mT: mesial temporal.
